# Development and Implementation of a “Music Beeps” Program to Promote Physical Fitness in Adolescents

**DOI:** 10.3390/ijerph17176148

**Published:** 2020-08-24

**Authors:** Hyun-Chul Jeong, Eui-Jae Lee, Hyun-Su Youn, Wi-Young So

**Affiliations:** 1 Department of Physical Education, Jeonbuk National University High School, Jeollabuk-do 54869, Korea; 01086445918@hanmail.net; 2 Department of Physical Education, Baek Seok High School, Goyang-si 10416, Korea; freedom_jae@hanmail.net; 3 Department of Physical Education, College of Education, Won Kwang University, Iksan-si 54538, Korea; 4 Sports and Health Care Major, College of Humanities and Arts, Korea National University of Transportation, Chungju-si 27469, Korea

**Keywords:** fitness promotion, high school students, music beeps (MP), physical activity, Physical Activity Promotion System

## Abstract

This study aimed to develop a physical education fitness program for adolescents to counteract the declining physical activity levels caused by the COVID-19 pandemic, as well as to investigate the program’s effect. This mixed-methods study developed and implemented a five-component “Music Beeps” (MB) program to promote adolescents’ physical fitness. A total of 240 students from two high schools in South Korea—divided into experimental and control groups—participated in 32 sessions over 16 weeks. The changes in students’ fitness were analyzed, and the educational effects were examined via inductive analysis of the observation logs and group and in-depth interviews. The results demonstrated that, whereas the comparison group demonstrated no statistically significant changes in power, muscular strength and endurance, or cardiopulmonary endurance, the experimental group showed changes in all these variables, along with changes in flexibility. Further, the MB program had significant educational effects. First, students reported that musical cues enhanced their fitness motivation and sense of responsibility. Second, record-keeping and active participation contributed to self-led fitness management. Third, activity in a small space with few pieces of equipment led to the positive perception that the program was efficient and enabled regular exercise regardless of climate conditions.

## 1. Introduction

Improving physical fitness through frequent physical activity and regular exercise is important in adolescence. However, in recent years, adolescents worldwide have mostly been sedentary in school and have been spending most of their leisure time on their cell phones. Further, due to the increased amount of food consumption in addition to reduced physical activity, the obesity rate is on the rise. It was found that adolescents have reduced fitness due to the use of electronic media [1], and Active Healthy Kids have mentioned the importance of fitness promotion programs for adolescents due to the low physical activity and fitness among children and adolescents [2].

The World Health Organization (WHO) recommends that children and youth aged between 5 and 17 years engage in at least an hour of moderate-to-vigorous-intensity physical activity (MVPA) daily for health benefits [3]. To address this issue, G7 countries, including the United States, Canada, and European countries, have implemented national policies for school physical education, which have been found to contribute to boosting students’ physique and fitness as well as significantly influence sociality and cooperation among students [4].

The “Adolescents’ physique and health status” report from 2000 to 2015 indicates that, while the obesity rate is consistently on the rise, muscle strength, muscle endurance, flexibility, and power are on the decline overall [5]. The reasons for a reduced fitness among adolescents include a poor diet, lack of sleep, and lack of physical activity. The Physical Activity Report for children and Adolescents in South Korea [6] reported that in 2018 adolescents’ fitness was rated as D+, indicating inadequate physical activity.

Along with reduced fitness, climate change has a diverse impact on school education and has induced various modifications. Physical education (PE) classes are limited because of fine dust, which is an emerging issue in the world; therefore, PE classes need to change due to environmental factors [7]. PE has emerged as a school subject that needs to remain sensitive to climate change, necessitating a new paradigm in education in response to such changes. Climate change has restricted and simplified PE, and such passive PE is believed to hinder the growth of adolescents in the future, ultimately taking a toll on their health and fitness.

The need to develop and implement more efficient fitness promotion programs for adolescents through school PE has been raised. The promotion of fitness programs that enable students to easily participate anywhere and anytime is necessary. Further, a program that can boost adolescents’ fitness within the areas available for PE at school needs to be developed and implemented [8,9]. Most existing fitness promotion programs either require teachers’ direct or indirect involvement or are not interesting enough to engage students. Therefore, a system in which these programs can be run efficiently with a minimal involvement of the teacher, while also engaging students, needs to be developed. In this context, this study aims to develop and apply a program that encourages self-directed fitness promotion among high school students to help them maintain and improve their health.

The purpose of this study was to investigate the effect and educational meaning of an MB fitness promotion program and to generalize the results in order to improve health and fitness promotion for students. This study was conducted in South Korea; a “Music Beeps” (MB) fitness promotion program was developed, which involved the use of cues in music tailored to students’ capabilities. The MB fitness promotion program utilizes musical cues to substantiate the potential of a more efficient fitness promotion program and examine its educational significance. This study’s implications will help provide alternative programs to encourage self-directed fitness management in adolescents.

## 2. Materials and Methods

### 2.1. Study Design

We planned and developed the fitness promotion program from September to December 2018 and administered 32 sessions of the program over 16 weeks from March to June 2019. The MB program was run for 20 min during a 50-min class twice a week, which included the time for warmup exercise; the experimental group participated in our MB program, while the comparison group underwent the exercise on its own. In other words, we compared the differences between the group that underwent fitness exercise according to musical cues and the group that underwent existing fitness exercise on its terms and examined the educational significance. The remaining portion of the PE class focused on badminton and volleyball, involving cooperative learning for each school.

This is a mixed-methods study, where the effects of the MB program were studied quantitatively, whereas its specific cases and educational significance were studied qualitatively. The use of this method is consistent with the emphasis on the need for qualitative research methodology in studies on adolescents’ health management [10,11]. Subsequently, to understand the complicated situations of adolescents and applying context-specific scientific programs based on this understanding, we chose a mixed-methods design. This facilitated the development of a highly efficient program based on an understanding of the strengths and shortcomings of an MB program.

This study was conducted following the principles stated in the Helsinki Declaration for studies with human subjects. Ethical approval from an institutional review board was approved by the Korea Ministry of Education and Korea Ministry of Culture, Sports, and Tourism (approval code: Jeollabukdo Office of Education 2019-081, date of approval: 2019-02-22), and informed consent was obtained from the participants.

### 2.2. Study Population

The participants were purposefully sampled from students in the tenth grade in one public and one national high school. From 240 students, 120 from each school, half (60 from each school, with 30 from each class) were non-randomly assigned to the experimental group and the rest to the comparison group. Table 1 illustrates the characteristics of the participants. These two schools collaboratively ran the fitness promotion program; to ensure the reliability of the program, the same curriculum was used for a semester for the two schools. The students were assigned to each group such that the mean physical activity promotion system (PAPS) values and the mean values for each factor were similar (±0.50) between the two groups.

For the deep-focus interviews, four students (one male and female student from each school) were selected from the experimental group. For both the deep-focus and group interviews, students who had indicated an interest and actively participated in PE class were selected. The reason for selecting only students who were active in PE was to identify the educational meaning of MB program participation for those who understood the purpose and content of the program well. Table 2 illustrates the characteristics of the interviewed students.

### 2.3. Instruments

#### 2.3.1. Physical Activity Promotion System

The Physical Activity Promotion System (PAPS) is a mandatory evaluation performed once a year on all elementary, middle, and high school students in South Korea. Power (50 m run and standing long jump), muscle strength and endurance (handgrip, sit-up, and push-up), flexibility (sitting trunk flexion), and cardiopulmonary endurance (shuttle run) are evaluated. Students are rated from 1 to 5 (1 is the highest, 5 is the lowest) for each category. The Korea Ministry of Education recommends that each school plans and runs fitness promotion programs for students rated 4 or 5, and all schools run a fitness program based on the PAPS results. In this study, we measured the essential PAPS evaluation items: 50 m run (power), sit-up (muscular strength and endurance), sitting trunk flexion (flexibility), and shuttle run (cardiovascular endurance). We took two PAPS measurements before and after the program on the same day to ensure that the results were not affected by the weather.

#### 2.3.2. Interviews

To examine in-depth the effects on students at each stage of the program described by Metz and Albers (2014), a semi-structured deep-focus interview was conducted [12]. The deep-focus interview, which examines the process of program participation from the planning and preparation stage, consisted in interviewing four participants ten times, for 30 min per session, immediately after the program. Following the individual deep-focus interviews, all the interviews were combined and analyzed to identify complementary and shared opinions.

### 2.4. Reliability and Research Ethics

We performed reliability testing for power, muscular strength and endurance, flexibility, and cardiopulmonary endurance parameters to ensure the reliability of the study. Reliability was tested using Cronbach’s alpha (α), which tests internal consistency; the results demonstrated that the reliability was very high for power (0.999), muscular endurance (0.997), flexibility (0.999), and cardiopulmonary endurance (0.995) in the comparison group and power (0.997), muscular endurance (0.992), flexibility (0.992), and cardiopulmonary endurance (0.991) in the experimental group. Consent was obtained from all participants before beginning the program. To ensure the trustworthiness, validity, and reliability of the study, we utilized member checking, peer debriefing, and triangulation. First, we reviewed whether the validity and meanings were well conveyed through a process in which the participants frequently checked the transcriptions, including the deep-focus interview transcriptions and observation logs. Further, peer debriefing was performed, in which one sports education professor, one sports education researcher (with experience in research pertinent to program development), and two sports education researchers (with experience in running fitness promotion programs) verified whether the data were interpreted correctly and the analyses were pertinent to the study’s aim and questions to ensure the study’s reliability. Finally, triangulation was performed for a consistent and reliable interpretation of the array of collected data. Moreover, we reviewed the analytical processes and study outcomes based on the collected data to determine whether our study was similar to previous studies and whether the validity and reliability of our study had been established.

### 2.5. Development and Staged Implementation of MB Program for Fitness Promotion

#### 2.5.1. Development of MB Program

In this study, the MB fitness promotion program was developed by adhering to the instructional design model based on the ADDIE model [13]. This is a systematic instructional design model for developing efficient programs in the field of education, which comprises five phases: analysis, design, development, implementation, and evaluation.

In the analysis phase—the first stage for developing a program—factors related to the program were analyzed. This phase involved analyzing the aims, techniques, and tasks of existing fitness promotion programs. We identified the need for a program that is both effective and fun for students as an alternative in response to the low motivation to participate in the existing fitness promotion programs and limited physical activity as a result of climate change. In the learner analysis, we analyzed the physical, cognitive, and affective domains of students; the students’ demands for a fitness promotion program were identified through interviews and a questionnaire. The tasks identified through the need and learner analyses were used to set the composition of the program, including goals, class plans, procedure, and evaluation. They were verified by one sports education professor, three individuals with research in educational programs, and five teachers who had run a fitness promotion program.

In the design phase, the specific purpose and goals of the program were set, and the criteria to assess goal achievement and instructional strategies for the program were designed based on the analyses in the previous phase. We designed the program to be fun and efficient using the restricted space and devices based on the learner and task analyses. Further, to ensure student-centered classes, class environments were selected through task posters and activity journals as opposed to teachers’ involvement.

In the development phase, the facility, device, and instructional materials for the program were developed according to the established goals. To secure instructional materials, class materials, facility, and the efficiency of the equipment to be utilized in the fitness program, we reviewed data, prepared the class environment, and examined learning experiences. The lesson plans and class model for each session of each program were finalized upon verification of their content validities by cooperating experts. Subsequently, a pilot 4-session program was administered to identify and address problems, based on which the program was finalized. We incorporated cues in music in stages to create a program tailored to the level of the learners and analyzed whether the cue for each stage was appropriate for the fitness of the students through a pilot experiment.

Based on the analysis of prior research, students, and students’ needs, we developed the MB fitness promotion program as follows. First, to ensure a student-centered program, the teachers’ roles were reduced to simple guidance, assistance, and encouragement toward the students for an active participation in the program. Second, the latest music and cues appropriate to students’ fitness levels were utilized to maintain participation and rest throughout the program. Third, the teacher presented the task and the students resolved the problem at hand, in which they tried to solve their personal or shared task. Instead of making the teacher guide the students, we designed the program such that students were able to cultivate their problem-solving abilities through various tasks.

#### 2.5.2. Implementation of the MB Program

The MB program developed in this study aimed to increase the fitness of adolescents and we established theoretical evidence for the purpose, contents, method, and evaluation of the program. In the development phase, we developed the contents for each session for the experimental and comparison groups and prepared instructional materials to be applied in each session. The program comprised 32 20-min sessions over 16 weeks (two sessions per week). Each component of the fitness promotion program was designed on the basis of the existing fitness programs verified in previous studies, and the reliability and validity of the program were established by fitness promotion program experts and the pilot test. As existing fitness promotion programs are perceived as uninteresting, we modified the music for the shuttle run and applied it to all programs to engage students and promote active participation. The shuttle run, also known as the 20 m Multi-stage Shuttle Run Test, is utilized to measure cardiopulmonary endurance in many countries [14]. A runner begins to run a 20 m distance with an initial speed of 8.5 km/h and a beep is set such that the interval between the beeps shortens progressively by 0.5 km/h. In South Korea, the latest K-pop music is played in between the beeps to engage students and promote participation. Thus, we edited the music and cues used in the shuttle run and applied them to all fitness promotion programs.

Based on the pre-study survey, we developed a five-stage program that corresponds to PAPS grades 1 to 5 with music files modified using Sound forge 8.0 (Sony Corporation, Tokyo, Japan) and Corel VideoStudio X3 (Corel, Ottawa, Canada). A total of 20 music files were used for the MB program. They were applied from stage 1; with the advancement to the next stage, its effectiveness was verified, and the level of improvement was examined via PAPS. The exercise program comprised power-muscular strength and endurance–flexibility–cardiopulmonary endurance components in order, with five minutes in each component in an indoor space (100 m^2^) at five stations (grade 1 to grade 5). Each session was equipped with minimal equipment, including a Bluetooth speaker (common), training ladder (power), yoga mat (muscular strength and endurance and flexibility), and jump rope (cardiopulmonary endurance). The students recorded their activities in a portfolio format in an activity log placed in the room and obtained feedback. To examine the efficiency of the cues in the music—the core component of the MB program—and its educational meaning, we used exercises that had been previously verified. To emphasize student-centered activity, teachers’ roles were minimized to guide students on the program and ensure safety. The fitness promotion program utilized musical cues for the participants to relieve stress from physical activity; their PAPS ratings were measured before and after the study to verify their improvement. To generalize the developed music and identify the amount of exercise from the name of the music file, the files were named according to the standard illustrated in Table 3. The name P1 5_50_10, 5_ represents total exercise duration (5 min), and 50_ and 10_ means exercise for 50 s and rest for 10 s. P1 indicates that the music is for the stage 1 power program.

#### 2.5.3. Implementation of the Power Program

In this study, we used plyometric training for the power program. Plyometric training is based on the fact that the quicker the muscles are stretched, the more power they exert; it has been reported to enhance significantly the power of various muscles [15]. Further, plyometric training is an excellent exercise for developing power, and it is the most widely used training worldwide to enhance power [16]. We designed the program with a focus on plyometric training but also to improve cardiopulmonary endurance, based on the effective characteristics of previous studies on the advantage of plyometric training [17,18]. The program involved repetitions of drills in the following order: right, left single leg forward jump on a training ladder–forward jump with both feet-right, left single leg side jump–two-feet jump from a 90-degree seated posture–right, and left single leg forward jump–two-feet side jump (left, right).

#### 2.5.4. Implementation of the Muscular Strength and Endurance Program

For the muscular strength and endurance program, we selected planks, which are frequently used in Tabata training. Plank exercise strengthens core muscles and it involves a modified push-up position on a yoga mat with the arms bent such that the arms and elbows make a 90-degree angle with only the toes, fingers, and the elbows touching the floor. We chose planks because previous studies confirmed that planks improve muscular strength and endurance and because we determined that holding the static posture would effectively improve muscular strength and endurance [19,20].

#### 2.5.5. Implementation of the Flexibility Program

We chose yoga postures for the flexibility program. In recent years, yoga has gained popularity as a form of fitness training and exercise, which increases muscular strength and flexibility; also, it has been reported that high school students enjoy yoga classes, and the subsequent health behaviors bring about benefits [21,22]. In this study, we used pranayama yoga poses in the following order: Garudasana (eagle pose), Janushirasana (standing head to knee pose), Ardha Matsyendrasana (spine twisting pose), and Padahastasana (hands to feet pose). The teacher explained the yoga poses in the task poster using pictures and descriptions, and students performed the yoga poses in order, according to their fitness level.

#### 2.5.6. Implementation of the Cardiopulmonary Endurance Program

For the cardiopulmonary endurance program, we chose jump ropes. Jump ropes have been proven to improve cardiopulmonary endurance [23,24]. Jump rope is a good exercise that produces maximum effects even in a small space, and it is easily accessible; it is easy to adjust the amount and intensity of exercise. In this study, we applied either two feet basic jump, two feet backward jump, or switching foot jump in each session.

### 2.6. Data Processing and Analysis

#### 2.6.1. Changes in the Degree of Fitness (PAPS)

To examine how the MB program for fitness promotion influenced students’ fitness, fitness was measured for the experimental and comparison groups before and after the program, and the obtained data were analyzed using the SPSS 24.0 software (IBM Corp., Armonk, NY, USA). Changes in the mean pre- and post-test scores within each group were analyzed using the paired sample *t*-test. The hypothesis of this study was that there would be significant differences in promotion of power, muscular strength and endurance, flexibility, and cardiorespiratory endurance between students who participated in the MB program and those who did not.

#### 2.6.2. Educational Significance of the Program

In this study, the educational significance of the MB program was explored via an inductive analysis of the data, involving transcription, coding, and categorization of expert discussions, observational logs, deep-focus interviews, and group interviews. First, we transcribed all collected data; then, while repeatedly reading and interpreting the transcriptions, we coded the data by their themes and recurring contents as related to the study question, conceptualizing the messages and meanings in the text.

## 3. Results

### 3.1. Evaluation of Each Fitness Component According to the Implementation of the MB Program

We divided the students into the experimental and comparison groups. We checked the experimental group’s power, muscular strength and endurance, flexibility, and cardiopulmonary endurance before the MB program (pre-test) and after the MB program (post-test) and analyzed whether they had statistically significant changes. The comparison group autonomously performed exercises for each component of the MB program, and their average changes were analyzed on the basis of pre- and post-test measurements. The results are illustrated in Table 4.

PAPS is a national evaluation system of student health and fitness that scores strength, muscular endurance, flexibility, cardiorespiratory endurance, and power. There was no statistically significant difference between the groups in the pre-tests for the dependent variables. In the comparison group, there were no statistically significant changes in power, muscular strength, or endurance and cardiopulmonary endurance, but there was a statistically significant change in flexibility. In the experimental group, there were significant changes in power at a 99% confidence level, with 9.041 in the pre-test and 8.886 in the post-test. There was also a statistically significant increase of mean muscular strength and endurance from 41.016 in the pre-test to 45.550 in the post-test. Further, there was a statistically significant increase in mean flexibility, from 8.764 in the pre-test to 9.595 in the post-test. Finally, there was a statistically significant increase in mean cardiopulmonary endurance, from 41.800 in the pre-test to 45.250 in the post-test.

### 3.2. Educational Significance Found through the Implementation of the MB Program

#### 3.2.1. Responsible, Music-Based Fitness Program

This study utilized fitness programs that have been validated in previous studies and added a modified version of the Shuttle Run music and cues to each program. Whereas existing fitness programs have involved the monotonous repetition of exercise or exercise while listening to music, musical cues used in the MB program prompt students to complete the given tasks with a sense of responsibility. Music enhanced the students’ interest and motivation and the beeper influenced them to be responsible for their fitness level.

*This fitness program was really helpful for me to exercise consistently because the cues in the music like the ones in the Shuttle Run signal me to do the target exercise and to rest.*

(Deep-focus interview with student B)

*It was definitely different from other fitness programs, where I exercised while listening to music. I think having to achieve the goal on cue gave me a sense of responsibility and set a clear goal to improve my fitness.*

(Deep-focus interview with student C)

Students participated in programs for grades 1–5 in each fitness component station. The goals for each grade, set depending on the level of improvement, gave students a sense of responsibility and motivation, which, in turn, served as an important factor that boosted students’ physical fitness.

#### 3.2.2. Self-Directed Health Management

Throughout the study, the students who participated in the MB program attempted to foster an ability to manage their health. Setting specific goals and keeping a record of their progress throughout the MB program provided them with a sense of responsibility and helped them practice self-led health management. This was one of the factors that actively engaged the students in the program. Setting goals according to the cues and keeping specific records to advance to the next stage allowed them to record their progress and difficulties, based on the feedback provided. Thus, the students could participate in the class and MB program more enthusiastically. The students could verify their fitness grades, and while continuously participating in the program, they learned about their level of fitness and improvement and attempted to achieve the goals they had set.

*I’m not very physically fit, and keeping a health practice report during this program gave me an opportunity to look at myself and learn the values of health. I became confident as I watched my records improve slowly.*

(Deep-focus interview with student A)

*I think adjusting the health learning goals that my teacher set for me to a level I can handle helped me a lot.*

(Deep-focus interview with student B)

*I really put myself into the program to achieve the fitness goals even without my teachers’ pushing me, and I think I developed good workout habits.*

(Student D’s fitness journal)

During the MB fitness promotion program, students developed an interest, as opposed to fear, in participating in an exercise to improve their health and actively participated in the program, without being bored, to achieve the shared goal of improving their fitness. As they experienced a new level of fitness and body images through various fitness promotion programs, they developed confidence, which, in turn, had a positive impact on their participation in other classes as well.

#### 3.2.3. Consistency and Simplicity of the Program

During our study period from March to June, fine dust, yellow dust, and heatwaves hit South Korea, and consequently normal schools had restricted PE classes. To overcome these climate conditions, we designed our program to utilize a small indoor space of about 100 m^2^ with only limited and simple equipment. In each station, we placed a Bluetooth speaker (common), training ladder (power), yoga mat (muscular strength and endurance and flexibility), and jump rope (cardiopulmonary endurance). The participants also perceived the efficiency of a small space that was not influenced by climate conditions and the use of only a few pieces of equipment as an advantage of the program:

*Last year, fine dust concentrations were high every day, so when we had combined classes with other classes in the auditorium, we were only able to do some simple warmups and couldn’t even play a single game. I had a hard time not being able to relieve stress because of the restricted PE classes, but I loved the idea of exercising efficiently in a small space with little time we have.*

(Deep-focus interview with student A)

*During the last year’s fitness program, we didn’t have enough squat equipment, and we had to move around all the time, so it was difficult to participate. But this time, no large equipment was involved, so it wasn’t hard preparing, and I was able to enjoy participating in the program.*

(Deep-focus interview with student C)

This program, which is not restricted by climate conditions or facilities, prompted students to participate in the MB program regularly, and with continuous participation, the students were able to see an improvement in their fitness.

## 4. Discussion

This study aimed to develop and implement a program to promote the physical fitness of adolescents through school PE classes to overcome the fact that adolescents are becoming less physically fit due to reduced physical activity worldwide. To this end, we utilized validated fitness programs to design an MB program that involved musical cues and compared them with self-directed fitness exercise. Ultimately, we attempted to verify the feasibility of an efficient fitness promotion program and examine its educational meaning.

The results demonstrated that the comparison group only showed statistically significant improvements in flexibility, whereas no significant changes were identified in power, muscular strength and endurance, or cardiopulmonary endurance. Conversely, the experimental group demonstrated statistically significant changes in power, muscular strength and endurance, flexibility, and cardiopulmonary endurance. In other words, the MB program improved power, muscular strength and endurance, flexibility, and cardiopulmonary endurance compared to existing fitness programs. This demonstrates the limitations of a fitness promotion program that relies on students’ autonomy. A teacher’s support has a great effect on a student’s psychological satisfaction and motivation and the music and beeps provided by the teacher to the students in this study were the results of students’ autonomous participation and responsibility [25]. As another study showed that fitness promotion programs should focus primarily on student participation, the positive results of the present study are likely due to the fact that students were able to do the activities whenever and wherever they desired with minimum teacher intervention, in order for the students to participate in the program actively [7,8]. In addition, it is thought that using internet-based wearables in fitness promotion program activities would improve motivation and responsibility [26].

As previous research revealed, the most important factor in fitness promotion to students is the utilization of a specific motivation strategy, this MB program differentiates itself from existing student fitness-related studies by being based on student autonomy and responsibility, which helped motivate students to participate in exercise [27]. The MB program is significant from an educational perspective. First, the cues that were modified along with music that attracted students’ attention served as a motivation for students to improve their fitness; while providing motivation, the program had value as a fitness promotion program that provided a sense of responsibility. As it is more effective to develop and apply youth physical strength improvement programs according to students’ preferences, the music and beeps in this study affected students’ interest and responsibility [28]. Second, as students kept their records and actively participated in class, they were able to engage in self-led management of their fitness and recognized the value and meaning of fitness. Third, activity in a small space with only a few pieces of equipment led to a positive perception of the program among the students as an efficient program that enabled regular exercise without being restricted by climate conditions. One of the reasons for the deterioration of students’ physical strength is the lack of specialized programs; however, the MB program applied in this study is an effective example of students’ physical strength improvement programs [29]. The results of this study can be used to develop physical activity school programs, as previous studies have reported that public health guidelines to increase the physical activity of adolescents should be created [30]. Finally, we hope that the MB program developed in this study serves as a platform for communication between students and PE teachers.

## 5. Conclusions

First, in this study, we have demonstrated the value of a program that can motivate students. Although the music and cues employed in this study were used only in fitness promotion programs, our findings suggest the possibility of applying them in a variety of other circumstances, such as regular PE classes or sports training. Utilizing music and cues in regular PE classes would motivate students and give them a sense of responsibility during class. This is because things that can motivate students within the limited time of the PE class help them become more deeply engaged in class and invest more effort.

Second, applications and programs that allow easy editing of music and cues for fitness promotion need to be developed. To ensure the efficiency of our program, support is required to help teachers manage music and cues more conveniently and utilize them in various educational environments.

Third, in this study, we separated the power, muscular strength and endurance, flexibility, and cardiopulmonary endurance stations. Examining the implementation of a mixed workout for circuit training would contribute to diversifying fitness promotion programs.

Fourth, this study was limited to general high school students in South Korea; thus, geographic location, environment, and socio-economic status of students’ families were not considered. It is expected that the MB program in this study will be applied in demographic experiments in other countries to add diversity.

## Figures and Tables

**Table 1:** Characteristics of the study population.

Group	Number of Participants (Male, Female)
Experimental group	30 male, 30 female students from a national high school
	30 male, 30 female students from a public high school
Comparison group	30 male, 30 female students from a national high school
	30 male, 30 female students from a public high school

**Table 2:** Characteristics of deep-focused interview participants.

Student	Sex	Characteristics
Student A	Male	His average PAPS rating is 3, which is similar to the class average. He has been interested in the fitness promotion program even before beginning the program, and he has shown vast interest in and actively participated in the program.
Student B	Female	Her average PAPS rating is 3, which is similar to the class average. She was not interested in the fitness promotion program before beginning the program but began to develop interest and actively participate in the program throughout the program.
Student C	Male	His average PAPS rating is 1 (highest level of fitness), and he has shown much interest in improving his fitness.
Student D	Female	Her average PAPS rating is 5 (low fitness), but she actively participates in PE class.

PAPS, physical activity promotion system; PE, physical education.

**Table 3:** Name of music file by exercise and meaning.

Component	Grade 1 (Excellent Fitness)	Grade 2 (Good Fitness)	Grade 3 (Moderate Fitness)	Grade 4 (Basic Fitness)	Grade 5 (Low Fitness)
Power (plyometric training)	P1 5_50_10	P2 5_40_10	P3 5_30_10	P4 5_20_10	P5 5_10_10
Muscular strength and endurance (Tabata) Planks	M1 5_50_10	M2 5_45_10	M3 5_40_10	M4 5_35_10	M5 5_30_10
Flexibility	S1 5_35_10	S2 5_30_10	S3 5_25_10	S4 5_20_10	S5 5_15_10
Cardiopulmonary endurance (Jump ropes)	C1 5_70_10	C2 5_60_10	C3 5_50_10	C4 5_40_10	C5 5_30_10

**Table 4:** PAPS fitness component evaluation for the experimental and comparison groups (*n* = 240).

Factor		*n*	Pre-Test		Post-Test		*t*-Value	*p*-Value
			Mean	Standard Deviation	Mean	Standard Deviation		
Comparison group	Power	120	9.053	1.374	9.036	1.371	1.936	0.055
	Muscular strength and endurance	120	41.041	18.990	41.341	18.784	−1.500	0.136
	Flexibility	120	8.757	6.331	8.837	6.192	−2.125	0.036 *
	Cardiopulmonary endurance	120	41.708	16.418	42.041	16.015	−1.609	0.110
Experimental group	Power	120	9.041	1.341	8.886	1.346	12.053	<0.001 ***
	Muscular strength and endurance	120	41.016	19.913	45.550	21.327	−13.281	<0.001 ***
	Flexibility	120	8.764	5.786	9.595	5.449	−9.256	<0.001 ***
	Cardiopulmonary endurance	120	41.800	16.421	45.250	16.234	−12.299	<0.001 ***

* *p* < 0.05, *** *p* < 0.001; tested by paired sample *t*-test.
